# *In silico* synchronization reveals regulators of nuclear ruptures in lamin A/C deficient model cells

**DOI:** 10.1038/srep30325

**Published:** 2016-07-27

**Authors:** J. Robijns, F. Molenberghs, T. Sieprath, T. D. J. Corne, M. Verschuuren, W. H. De Vos

**Affiliations:** 1 Laboratory of Cell Biology and Histology, Department of Veterinary Sciences, University of Antwerp, Antwerp, Belgium; 2 Cell Systems and Imaging Research Group (CSI), Department of Molecular Biotechnology, Ghent University, Ghent, Belgium

## Abstract

The nuclear lamina is a critical regulator of nuclear structure and function. Nuclei from laminopathy patient cells experience repetitive disruptions of the nuclear envelope, causing transient intermingling of nuclear and cytoplasmic components. The exact causes and consequences of these events are not fully understood, but their stochastic occurrence complicates in-depth analyses. To resolve this, we have established a method that enables quantitative investigation of spontaneous nuclear ruptures, based on co-expression of a firmly bound nuclear reference marker and a fluorescent protein that shuttles between the nucleus and cytoplasm during ruptures. Minimally invasive imaging of both reporters, combined with automated tracking and *in silico* synchronization of individual rupture events, allowed extracting information on rupture frequency and recovery kinetics. Using this approach, we found that rupture frequency correlates inversely with lamin A/C levels, and can be reduced in genome-edited *LMNA* knockout cells by blocking actomyosin contractility or inhibiting the acetyl-transferase protein NAT10. Nuclear signal recovery followed a kinetic that is co-determined by the severity of the rupture event, and could be prolonged by knockdown of the ESCRT-III complex component CHMP4B. In conclusion, our approach reveals regulators of nuclear rupture induction and repair, which may have critical roles in disease development.

The nuclear envelope is the principal barrier dictating bidirectional communication between the nucleus and cytoplasm of the cell. Directly underneath the lipid bilayer resides a dense meshwork of intermediate filaments, the nuclear lamina, which provides structural support for the nucleus and has a central role in nuclear organization and gene regulation^1^. Defects in one of its major protein constituents, the A-type lamins, cause a broad spectrum of tissue-specific and systemic diseases collectively referred to as laminopathies. Disease manifestations include muscular dystrophies, lipodystrophies and the premature aging syndrome Hutchinson-Gilford Progeria (HGPS).

Several hypotheses have been proposed to explain disease development at the cellular level. These are based on either the involvement of lamins in maintaining the mechanical integrity of the nucleus or their role in modulating transcription and signalling pathways by serving as docking sites for regulatory proteins^1^. Recently, we discovered a novel mechanism that unites aspects of both aforementioned hypotheses, namely temporary loss of nuclear compartmentalization due to ruptures of the nuclear envelope, causing inappropriate exchange of components between the cytoplasm and the nucleus^2^,^3^. Ruptures occur at weak spots of the nucleus, i.e. protrusions and regions devoid of lamins, pointing to mechanical defects, while the uncontrolled translocation of transcription factors during those events alter gene expression programs^2^. Moreover, ruptures are not only accompanied by transient shifts in regulatory protein distribution, but also seem to provoke more permanent translocations of macromolecular complexes (e.g. of PML bodies)^2^,^3^. Nuclear ruptures have also been observed in viral infections, where they are considered to represent hallmarks of nuclear entry and/or egress^4^,^5^. Since similar defects in nuclear compartmentalization have recently also been described in aging and cancer cells^6^,^7^,^8^,^9^ – both associated with abnormal expression of lamins or their precursors^10^,^11^ – it most likely represents a pathophysiological mechanism with generic relevance.

As yet, not much is known about the exact causes of nuclear ruptures, or about the specific functional consequences for the cell. It has been shown that growing cells on soft substrates reduces rupture frequency^12^, and cell confinement promotes rupture incidence^9^, suggesting involvement of the cytoskeleton. However, deregulated phosphorylation by protein kinase C family members has also been proposed as a potential causative mechanism^4^. Considering the consequences, it is important to note that rupture-prone cells do not die. On the contrary, even after repetitive rupture, cells continue to divide^2^, which implies they are able to repair the damaged nuclear envelope. Pinpointing the exact processes that precede, accompany or directly follow nuclear rupture is essential to better understand disease progression and to reveal novel biomarkers or targets for therapeutic interventions. Unfortunately, studying the causes and consequences of spontaneous nuclear ruptures is hampered by their stochastic nature and variable frequency. Here we describe a quantitative approach to study nuclear rupture induction and repair in a systematic manner. Using this approach, we revealed novel regulators of rupture events.

## Results

### Robust quantification of nuclear rupture events

**Figure 1:**
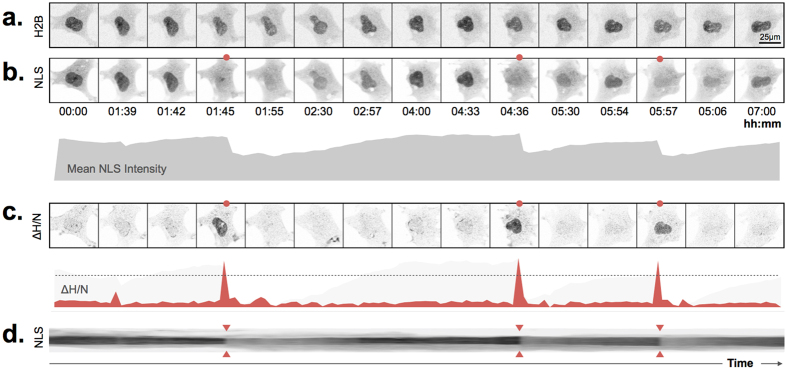
Robust detection of nuclear ruptures. MEF-LKO cell transfected with (**a**) H2B-GFP and (**b**) mCherry-NLS demonstrating repetitive nuclear ruptures (time point of ruptures marked by red dots) as witnessed by a sudden decrease in average intensity of the nuclear signal; (**c**) the temporal derivative of the H2B/NLS signal ratio (ΔH/N) shows a strong concurrent increase. The dotted line indicates the cutoff value, above which ruptures are detected; (**d**) kymograph of the full temporal recording of the NLS channel (arrowheads indicate rupture events).

Nuclear ruptures are characterized by temporary loss of nuclear compartmentalization. This can be visualized by transient relocation of fluorescently labeled nuclear proteins to the cytoplasm^2^. A convenient marker that is relatively inert with respect to nuclear function and readily translocates during nuclear ruptures is a fluorescent protein coupled to a nuclear localization signal. Indeed, when monitoring mCherry-NLS during nuclear ruptures, the nuclear signal dramatically decreases (Fig. 1b,d; Suppl. Movie 1). When nuclear signals restore quickly, i.e. within 10–15 min, and cells are not very mobile, nuclei can be tracked automatically solely based on the mCherry-NLS signal by allowing temporal gaps in the tracking algorithm (Suppl. Fig. S1; Suppl. Movie 2). However, quite often recovery of the nuclear signal takes much longer, precluding proper track assignment. In addition, highly mobile cells may temporarily move out of focus, causing transient decreases in nuclear intensity, which are not related to rupture events and therefore add noise to the detection process. To bypass these problems, we co-expressed a marker that is not translocated during rupture events, H2B-GFP, and to which NLS signals were normalized (Fig. 1a). When a rupture occurs, the nuclear NLS signal drops, while the H2B signal remains constant, causing an increase in the H2B/NLS ratio. As such, ruptures are robustly detected when the derivative of the H2B/NLS signal ratio (ΔH/N) exceeds a fixed threshold value, corresponding to a >20% decrease in nuclear NLS signal (Fig. 1c; Suppl. Movie 1).

### Rupture frequency correlates with nuclear deformation in MEF-LKO cells

**Figure 2:**
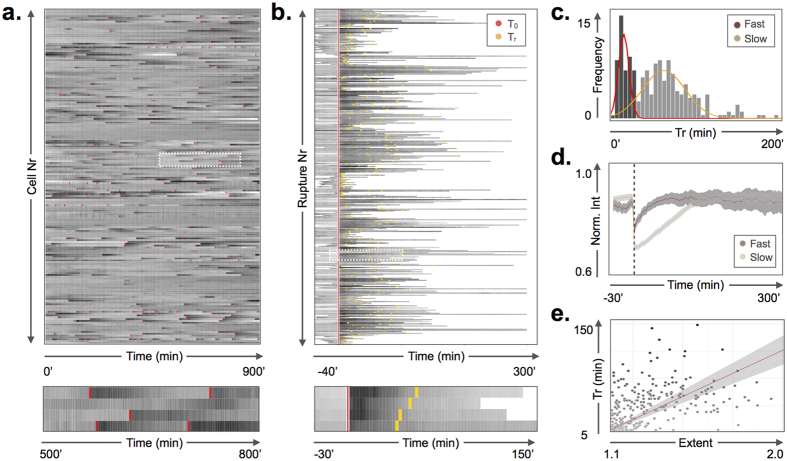
*In silico* synchronization of nuclear ruptures. (**a**) Time tracks of individual MEF-LKO cells (n = 216 tracks) showing fluctuations in nuclear intensity and multiple rupture events (red marks). Magnified view of rectangular selection below; (**b**) Individual rupture events extracted from all time tracks synchronized to the moment of nuclear rupture (red) and marked with recovery halftime (yellow). Magnified view of rectangular selections below. (**c**) Histogram of recovery halftimes, superimposed with the individual components of the best-fit bimodal density distribution showing two distinct populations corresponding with fast (red) and slow (orange) recovery; (**d**) bimodal recovery kinetics of nuclear signal after rupture, represented as average normalized signal ± standard error (shaded ribbon) of fast (T_r_ ≤ 30 min) and slow populations (T_r_ > 30 min). Black dotted line indicates moment of rupture; The Y-axis has been cropped for clarity; (**e**) Scatterplot with linear fit (R^2^ = 0.519) and 99% confidence interval showing that recovery halftime partially scales with the severity of rupture (expressed as extent of nuclear intensity drop).

**Figure 3:**
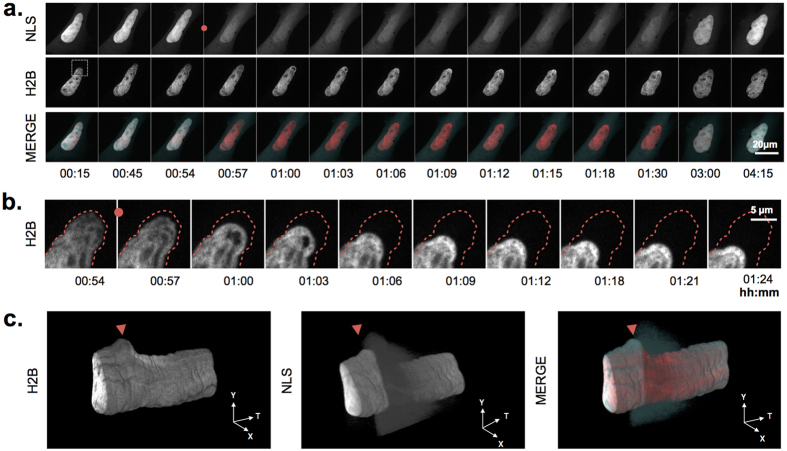
Local chromatin condensation during rupture. (**a**) Montage of a MEF-KO cell, transfected with H2B-GFP and NLS-mCherry, showing pronounced nuclear deformation after nuclear rupture (red dot) and adjoined chromatin condensation, as shown by the concomitant increase in H2B signal intensity. Selected time points of individual channels are shown in grayscale and as merge with the NLS channel in cyan and the H2B channel in red; (**b**) Magnified view of the rectangular region indicated in (**a**) superimposed with outlines (dotted red line) of the pre-rupture nuclear shape (00 h:54 m); (**c**) XYT kymographs (rendering of a single confocal section through time) showing the pronounced deformation after nuclear rupture (red arrowhead) and the subsequent local increase in H2B signal intensity.

Using this co-expression system, we acquired time-lapse image data sets of *Lmna* knockout mouse embryonic fibroblasts (MEF-LKO), which have previously been shown to experience nuclear ruptures^2^. Figure 2a shows the intensity profiles of ~200 cells that were tracked throughout a 15 h time frame. On average, approximately two ruptures occurred per tracked nucleus, with a maximum of 15 ruptures in a single cell. However, 45% of the nuclei did not experience ruptures within this time frame, almost doubling the actual rupture frequency for those cells that are rupture-prone. Interestingly, rupture-prone cells showed significantly more nuclear deformation (measured as nuclear circularity fluctuations^13^) than MEF-LKO cells that did not undergo ruptures (Suppl. Fig. S2a,b). In line with this, we frequently observed local contractions of the nucleus after severe rupture events, i.e. those with a strong NLS intensity decrease and long recovery time. Such contractions were accompanied by focal chromatin condensation, as evidenced by temporarily increased H2B signal intensity (Fig. 3; Suppl. Movie 3).

### Differential rupture recovery kinetics in MEF-LKO cells

Rupture events for which the nuclear NLS signal restored completely to the pre-rupture level within the imaging time frame were synchronized *in silico* to analyze the recovery kinetics (Fig. 2b). The average recovery halftime (T_r_) for MEF-LKO cells was 54 ± 44 min. When put under scrutiny, the recovery halftime showed a bimodal histogram (Hartigan’s dip test: p < 0.05), pointing to the presence of two populations (Fig. 2c). Unsupervised clustering based on maximum likelihood estimation identified a population with fast (T_r_ = 13 ± 6 min) and one with slow (T_r_ = 67 ± 43 min) recovery halftime (p < 0.01). The tipping point, i.e. the value with equal probability of belonging to one of both populations, was 30 min. The recovery halftime roughly scaled (R^2^ = 0.519) with the magnitude of the intensity drop witnessed directly after rupture, suggesting that the recovery at least in part correlates with the severity of the rupture event (Fig. 2e). However, the initial rise of the recovery curve showed a significantly steeper slope (p < 0.001) for the fast population (T_r_ ≤ 30 min) than for the slow population (T_r_ > 30 min), pointing to the presence of additional determinants (Fig. 2d). Notably, nuclei that ruptured repetitively showed a higher average recovery halftime than those that ruptured only once within the same time frame (Suppl. Fig. S2c).

### *LMNA* depletion increases nuclear rupture frequency in human model cells

**Figure 4:**
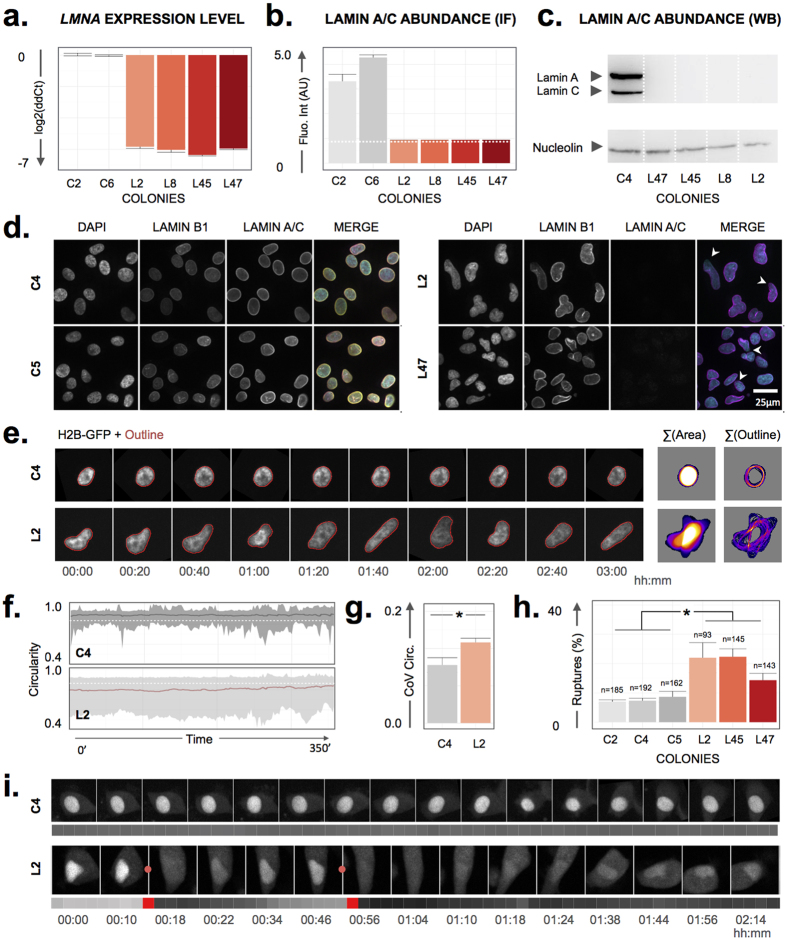
HT-LKO cells recapitulate the hallmarks of lamin A/C deficiency. (**a**) Quantitative PCR shows a >6-fold reduction (expressed as log_2_ fold change of ddCt value) of *LMNA* transcripts in different LKO colonies as compared to HT-WT clones; (**b**) Quantitative immunofluorescence shows a dramatic reduction of A-type lamin levels in HT-LKO colonies, approximating background levels (dotted white line); (**c**) Western blot for lamin A/C reveals the absence of both proteins in HT-LKO cells; (**d**) Immunostaining and nuclear counterstaining of HT-LKO cells reveals their aberrant nuclear morphology, virtual absence of lamin A (green) and local depletion of lamin B (red) (arrowheads) as opposed to HT-WT cells; (**e**) Time-lapse recordings after H2B-GFP transfection illustrate increased nuclear plasticity of HT-LKO vs. HT-WT cells, as evidenced by their larger projected area (Σ Area) and contour changes (Σ Outline) across time; (**f**) HT-LKO nuclei have a lower average nuclear circularity (dotted white line indicates a circularity of 0.85) and higher nuclear circularity fluctuations (average + 95% confidence interval) than HT-WT cells; The Y-axis has been cropped for clarity; (**g**) This translates into a significantly larger coefficient of variation (CoV) for the circularity across time (p < 0.001). (**h**) Nuclear ruptures occur more frequently in HT-LKO cells than in HT-WT cells (p < 0.001); (**i**) Representative montages of the NLS channel of a C4 HT-WT and L2 HT-LKO cell, with corresponding NLS/H2B ratio measurements (grey-coded bar plots). The moments of rupture events are indicated as red dots. Bar graphs reflect mean ± standard error (n = number of tracks).

To ascertain that nuclear rupture events are truly caused by *LMNA* deficiency in human cells, we established *LMNA* knockout human HT-1080 cell lines (HT-LKO) using targeted CRISPR/Cas9 genome editing. Multiple clones were isolated and compared with control-treated, wild type cells (HT-WT), which underwent identical operational procedures but without the specific guide RNA. All selected HT-LKO colonies demonstrated ≥6-fold reduction of *LMNA* transcripts and virtual absence of lamin A/C proteins (Fig. 4a–c) as compared to HT-WT clones. Monoclonal HT-LKO cells recapitulated the hallmarks of lamin A/C deficiency^13^, including nuclear dysmorphy, local depletion of B-type lamins and increased nuclear plasticity (Fig. 4d–g). While HT-WT cells ruptured infrequently (5–10%), HT-LKO clones experienced significantly more spontaneous rupture events (p < 0.0001), up to 40% in a 3 h time span (Fig. 4h,i). Nuclear ruptures were not lethal (Suppl. Fig. S3a; Suppl. Movie 4), and were accompanied by local nuclear deformation, sometimes followed by temporary chromatin condensation as witnessed in MEF-LKO cells (Suppl. Fig. S3b–d). On average, nuclear signal recovery in HT-LKO was much faster than in MEF-LKO cells (T_r_ = 23 ± 35 min for HT-WT; T_r_ = 23 ± 30 min for HT-LKO) and the bimodal recovery kinetics were not observed (Suppl. Fig. S4). Using the same CRISPR/Cas9 genome editing approach, we also established *ZMPSTE24* knockout HT-1080 cell lines (HT-ZKO) (Suppl. Fig. S5). The ZMPSTE24 metalloprotease is responsible for the final cleavage step in the posttranslational maturation of lamin A, and loss of this enzyme induces accumulation of farnesylated prelamin A^14^. Despite a variable, clone-specific effect on *ZMPSTE24* transcripts (which is no prerequisite for efficient knockout), all HT-ZKO cells demonstrated a lack of mature lamin A, strong accumulation of prelamin A (Suppl. Fig. S5b,c) and overt nuclear dysmorphy (Suppl. Fig. S5c). However, we did not observe a significant increase in rupture frequency as compared to HT-WT controls (Suppl. Fig. S5c).

### Experimental modulation exposes regulators of rupture induction and repair

**Figure 5:**
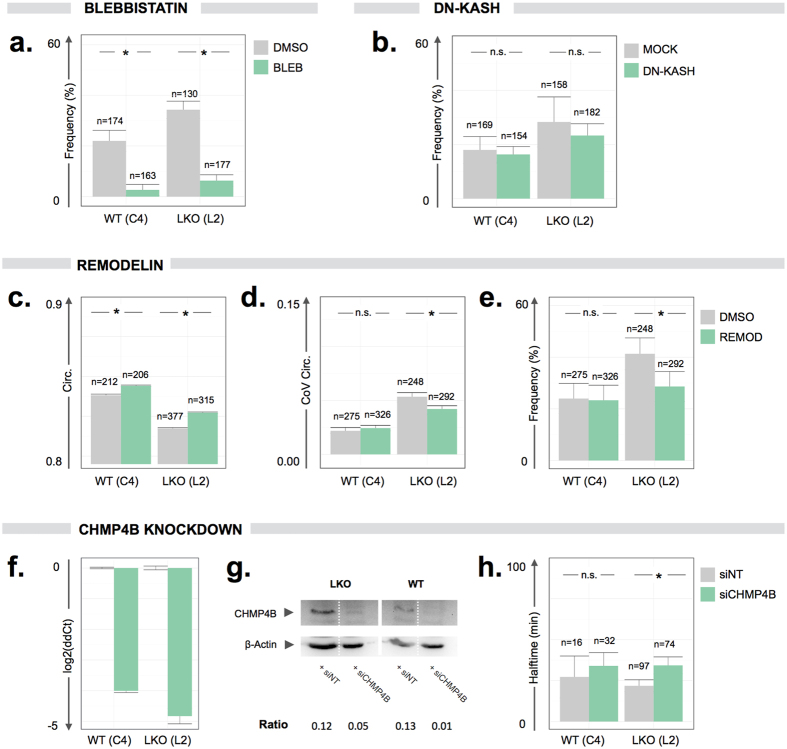
Nuclear rupture kinetics can be experimentally modulated. (**a**) Blebbistatin (BLEB) reduces rupture frequency in both L2 HT-LKO and C4 HT-WT cells (p < 0.001); (**b**) Expression of a dominant-negative KASH construct (DN-KASH) does not significantly affect rupture frequency in L2 HT-LKO or C4 HT-WT cells; (**c**) Remodelin (REMOD) significantly increases the circularity (p < 0.001) in L2 HT-LKO or C4 HT-WT cells. The Y-axis has been cropped for clarity; (**d**) Remodelin significantly reduces the coefficient of variation (CoV) for the circularity across time L2 HT-LKO cells (p < 0.01) but not in C4 HT-WT cells; (**e**) Remodelin reduces rupture frequency in L2 HT-LKO cells (p < 0.05) but not in C4 HT-WT cells; (**f**) siRNA-mediated knockdown of *CHMP4B* (siCHMP4B) causes >95% reduction (expressed as log_2_ fold change of ddCt value) of *CHMP4B* transcript levels as compared to the non-targeting control siRNA (siNT); (**g**) siRNA-mediated knockdown of *CHMP4B* causes >50% reduction of CHMP4B protein levels as compared to the non-targeting control siRNA; (**h**) siRNA-mediated knockdown of *CHMP4B* increases nuclear recovery halftimes (p < 0.05). Bar graphs reflect mean ± standard error (n = number of cells).

Since all HT-LKO colonies experienced significantly increased rupture frequency with comparable recovery halftimes (Fig. 4, Suppl. Fig. S4), subsequent experiments were performed with one HT-LKO (L2) and HT-WT (C4) clone. Since cells grown on soft substrates experience less ruptures^12^ and confinement increases rupture incidence^9^,^15^, mechanical forces exerted by the cytoskeleton are presumed to play an important role in nuclear rupture events. To test this hypothesis, we selectively inhibited actomyosin contractility by blebbistatin. This significantly reduced rupture frequency in HT-LKO cells as well as HT-WT cells (Fig. 5a). Since actin microfilaments are anchored to the nuclear lamina through the LINC complex, we assessed whether perturbing this connection using a dominant negative KASH construct (DN-KASH)^16^ affected rupture incidence. However, whilst effectively displacing endogenous nesprins (Suppl. Fig. S6), DN-KASH overexpression did not cause a significant change in rupture incidence in HT-LKO and HT-WT cells (Fig. 5b).

Given the overt nuclear dysmorphy and nuclear plasticity in HT-LKO cells (Fig. 4e–g), we wondered whether normalization of nuclear shape could alleviate the rupture phenomenon. To this end, we exposed HT-LKO cells to the NAT10 inhibitor remodelin, which has been shown to normalize nuclear shape in *LMNA* knockdown cells and Progeria patient cells^17^. Remodelin significantly (p < 0.001) increased nuclear circularity in HT-WT and in HT-LKO cells (Fig. 5c), suggesting normalization of nuclear shape, and it specifically reduced nuclear circularity fluctuations across time in HT-LKO and MEF-LKO cell types (Fig. 5d, Suppl. Fig. S7a), suggesting a decrease in nuclear plasticity. In line with this, remodelin treatment significantly (p < 0.05) reduced rupture frequency in both HT-LKO cells and MEF-LKO cells, but not in HT-WT cells (Fig. 5e, Suppl. Fig. S7b).

Recovery of the nuclear signal after rupture implies that the barrier function of the nuclear envelope is restored. Since resealing of a broken membrane is a thermodynamically inefficient process – especially when it involves large gaps^18^ – a repair machinery may facilitate nuclear envelope repair. Since the ESCRT-III complex has recently been suggested to play a key role in nuclear envelope quality control and repair^9^,^15^,^19^,^20^, we selectively knocked down *CHMP4B*, a critical component of the ESCRT-III complex (Fig. 5f). Knockdown resulted in a consistent 95% decrease of *CHMP4B* mRNA levels and 60–90% decrease in protein levels with respect to a non-targeting control (Fig. 5g). After *CHMP4B* knockdown, we found a significant (p < 0.05) increase in T_r_ in HT-LKO cells but not in HT-WT cells (Fig. 5g).

## Discussion

Nuclear rupture is an emerging hallmark of various pathologies, including laminopathies, viral infection and cancer^2^,^5^,^7^. In this work, we presented a robust approach for interrogating nuclear rupture kinetics in a systematic and quantitative manner. In contrast with previously described methods to gauge nucleocytoplasmic shuttling^21^, our approach relies on an internal reference – a tightly bound histone protein – that covers for long-term loss of compartmentalization and focus fluctuations. This proved to be essential, especially for accurately tracking and measuring HT-LKO cells, which are notoriously mobile^22^. Individual rupture events were readily detected by differentiating the H2B/NLS ratio as a function of time, and they were synchronized *in silico* to determine their recovery kinetics.

We first validated the approach in MEF-LKO cells. We found that the automatically determined rupture frequency of ~55% aligned well with previously performed manual quantifications^2^. We also observed increased nuclear plasticity in rupture-prone cells. It is conceivable that this subset of cells experiences increased cytoskeletal forces on their nuclei, causing them to rupture more easily. Alternatively (or additionally), the increased plasticity may be a reflection of the local contractions of the nucleus that follow spontaneous rupture events. Since we have only acquired single confocal sections, we can not exclude that local Z-axis torsions occur, but the extent of deformation and the fact that fibroblasts on a hard substrate have very flat nuclei even in the absence of A-type lamins (5–7 μm height)^23^,^24^, strongly supports lateral contractions. The contractions were often accompanied by focal chromatin condensation, triggered at the site of rupture. Chromatin condensation and nuclear shrinkage have previously been reported to precede apoptotic events^25^. However, we have shown that nuclear ruptures are not lethal per se. Chromatin compaction may occur in a regulated manner independent of apoptosis, for instance by local remodeling of chromatin compaction-inducing proteins such as NET23^26^, as a result of local nuclear membrane loss and/or replenishment. Alternatively, since chromatin condensation has been associated with migratory phenotypes^27^ and chromatin rapidly remodels upon external force application^28^,^29^,^30^, it may represent a protective response towards a mechanical insult. In effect, it resembles the compaction observed after mechanical stimuli, which have been attributed to ATR-mediated disengagement of chromatin from the nuclear envelope^31^.

The severity of rupture events in MEF-LKO cells varied both in terms of extent (relative signal decrease) and recovery halftime. Assuming that the recovery halftime reflects a combination of two processes, namely repair of the damaged nuclear envelope and re-import of cytoplasmic GFP-NLS, one could argue that more severe ruptures, i.e. those with stronger nuclear signal losses, automatically lead to larger recovery halftimes. For, larger punctures cause more leakage and might be more difficult to repair. But, while we indeed found a positive correlation between rupture extent and recovery halftime, the correlation was far from perfect: many rupture events with a modest extent still showed a larger recovery halftime. In addition, fast and slow populations were typified by different initial recovery rates, reflecting differential import behavior. This implies that the nuclear envelope is not always easily resealed after rupture, irrespective of the severity of the event, or that simultaneous leakage slows down signal restoration. Such a severe state of compromised nuclear compartmentalization may be attributed to the occurrence of additional (weak, sub-threshold) ruptures and/or generalized increased nuclear permeability. Favoring this hypothesis, nuclei that ruptured repetitively showed a higher average recovery halftime than those that only ruptured once within the given time frame. In this respect, we also note that local depletion of nuclear envelope components, such as B-type lamins and nuclear pore complexes, is not omnipresent in a population of MEF-LKO cells, plausibly revealing a fraction of more susceptible cells^32^. A general reduction of nuclear pore complexes may further reduce nuclear import, as also observed after overexpression of laminopathy-causing prelamin A forms^33^,^34^.

MEF-LKO cells have been extracted from a single mouse^32^ and lack isogenic controls, which is why we cannot rule out cell- or genotype-specific effects. To guarantee controlled evaluation of A-type lamin deficiency, we therefore established human *LMNA* knockout cell lines (HT-LKO) and genotype-matched controls (HT-WT). In contrast with normal (*LMNA*^+/+^) fibroblasts - in which we had never observed spontaneous nuclear ruptures^2^ - HT-WT cells displayed spontaneous rupture events, albeit at low frequency. Nevertheless, we found HT-LKO cells to clearly recapitulate the hallmarks of lamin A/C deficiency, including nuclear dysmorphy, nuclear plasticity^13^,^35^, and most importantly, increased rupture frequency. The latter suggests that absence of A-type lamins indeed increases nuclear fragility. Mere absence of mature lamin A is not a prime determinant since rupture frequency was not significantly increased in *ZMPSTE24* knockout cells (HT-ZKO). HT-ZKO cells still produce normal levels of lamin C, which may balance the lack of mature lamin A. Indeed, lamin A and lamin C have been shown to regulate nuclear mechanics and nuclear stiffness equally well^36^. While the accumulation of prelamin A in these cells may further enhance nuclear stiffness – thus exerting a protective effect to ruptures – it may also render them more vulnerable to mechanical strain, as observed in HGPS patient cells, which accumulate a farnesylated form of prelamin A, termed progerin^37^. Thus, prelamin A might have an ambivalent role. In previous work we found that fibroblasts from patients suffering from HGPS and restrictive dermopathy demonstrate much less ruptures than lamin A/C deficient fibroblasts, but still significantly more than control fibroblasts from healthy individuals^2^. However, HT-WT cells already demonstrate ruptures by default, whereas normal fibroblasts do not. This may also explain the lack of detectable difference between HT-WT and HT-ZKO cells.

We next probed for potential rupture-inducing mechanisms. Given the putative role of the cytoskeleton, we first blocked actomyosin contractility using blebbistatin. This treatment dramatically reduced nuclear rupture frequency in both HT-WT and HT-LKO cells. This aligns well with recent findings on actomyosin-driven ruptures in cells migrating through confining spaces^9^ and explains why soft substrates – which are also presumed to reduce actomyosin tension^38^ - reduce rupture frequency^12^. In many adherent cells, the nucleus is shaped by an apical actin cap composed of stress fibers, which is physically connected to the nuclear envelope through linkers of nucleoskeleton and cytoskeleton (LINC) complexes^39^,^40^. We therefore tested whether disruption of these connections using a truncated nesprin construct (DN-KASH), which competitively binds SUN domain proteins^16^, could alter rupture frequency. However no significant effect was observed. Since A-type lamins are major intra-nuclear binding partners of nesprins, it is highly plausible that both LINC complexes and the actin cap are severely perturbed in HT-LKO cells^39^,^41^,^42^, thus explaining the lack of effect in these cells. The insensitivity to DN-KASH in HT-WT cells on the other hand, seems to suggest that neither the LINC complex nor the actin cap play a major role in spontaneous rupture induction. In line with this, it has recently been shown that the LINC complex is not required for maintaining a flat nuclear shape^23^,^38^, thus pointing towards the involvement of indirect compressive or tensile forces, possibly mediated by lateral actin fibers. In addition, it has been shown that loss of nesprins 1 and 2 can trigger typical features of nuclear dysmorphy and compromised nuclear envelope integrity^43^, thereby arguing against a causal role in rupture induction.

Hallmarks of lamin A/C deficiency include nuclear dysmorphy and nuclear plasticity. When using the selective shape-normalizing compound remodelin^17^, we found a marked decrease in nuclear rupture incidence in both MEF-KO and (to a lesser extent) HT-KO cells, which correlated with a significant decrease in nuclear plasticity. Remodelin targets the acetyl-transferase NAT10, which has been proposed to regulate microtubule stability^44^. Microtubules exert compressive forces as well as indirect tensional forces (via dynein^45^) on the nucleus and it has also been shown that microtubule polymerization induces nuclear envelope folding in interphase cells^46^. Since lamin A/C-deficient cells have fragile nuclei^35^, they may be more susceptible to such forces than control cells with more rigid nuclear envelopes. However, we cannot rule out whether the effect is solely mediated by microtubule reorganization or also involves functions related to the histone acetylation activity of NAT10^47^ or its responsiveness towards oxidative stress^47^ – yet another hallmark of lamin deficiency^48^,^49^.

Finally, nuclear rupture is non-lethal and nuclear intensity recovers, suggesting the involvement of repair machinery. The ESCRT-III complex has been shown to play an essential role in nuclear envelope resealing during late stages of cell division and its depletion disrupts nuclear envelope integrity^20^,^50^. In line with findings that were published during revision of this manuscript^9^,^15^, depletion of the ESCRT-III component CHMP4B, increased recovery times significantly, highlighting the pivotal role of this complex, not only for mitotic but also for interphase surveillance of nuclear envelope integrity.

In conclusion, we have established a robust approach for quantifying nuclear rupture kinetics with which we have revealed novel regulators of rupture induction and repair. Scrutinizing the driving forces behind these events will further our understanding of the rapidly expanding tree of laminopathies, and may help identifying new therapeutic entry points for an even broader spectrum of disorders in which the nuclear barrier function has become compromised.

## Materials and Methods

### Cell Culture

Normal (MEF-WT) and *Lmna* knockout mouse embryonic fibroblasts (MEF-LKO) and human fibrosarcoma cells (HT-1080, kindly shared by Prof. K. Wolf, Radboud University of Nijmegen, The Netherlands) were cultured in DMEM high glucose with L-glutamin (Lonza, BE12-604F/12) supplemented with 10% fetal bovine serum (Gibco, 10270-106), and 1% penicillin/streptomycin (Gibco, 15140-122), according to standard procedures. Proliferative capacity was monitored by cell counting with every passage and cultures were tested for mycoplasma infection using a PCR test kit (Bio-connect, PK-CA91-1024) every two months.

### Compound treatment

Cells were treated with remodelin (VWR, CAYM16066-1), 24 h before live cell imaging. Remodelin was supplied in DMEM with supplements at a 50 μM concentration. Before live cell imaging the medium was refreshed with the same dose of remodelin. Blebbistatin treatment was started one hour before live cell imaging at a concentration of 100 μM. Controls were supplied with the same amount of DMSO as was present in the treated cells and was always ≤0.5% of the total volume.

### Transfection

The following constructs were used: mCherry-NLS, H2B-GFP (both generous gifts from Dr. J. Goedhart, University of Amsterdam, the Netherlands) and DN-KASH-EGFP (a generous gift from Prof. G. Gundersen, Columbia University, New York^16^). For transfection of expression plasmids, Lipofectamine 2000 (Life Technologies, 11668027) was used according to the manufacturer’s instructions. siRNA mediated knockdown of *CHMP4B* (Dharmacon, M-018075-00-0005) was executed with lipofectamin RNAiMAX (Life technologies, 13778075) according to the manufacturer’s instructions with the lowest siRNA concentration (5 nM) that elicited 95% downregulation. Stealth RNAi siRNA Negative Control, Med GC (Life Technologies, 12935-300) was used as a negative control as it is not homologous to and therefore does not target any vertebrate sequence. After 72 h cells were subjected to live cell imaging.

### Genome editing

To obtain stable knockout HT1080 cell lines for *LMNA* (HT-LKO), *ZMPSTE*24 (HT-ZKO) and controls (HT-WT), we used CRISPR-Cas9 genome editing. The gRNA and Cas9 protein were delivered to cells by plasmid transfection. The plasmids were constructed starting from pSpCas9(BB)-2A-GFP (PX458) or pSpCas9(BB)-2A-Puro (PX459) (from Feng Zhang, Addgene # 48138 and 48139). The gRNA sequence which targets the first exon of the gene was: 5′-CCTTCGCATCACCGAGTCTGAAG -3′ for *LMNA* and 5′-GGCCGAGAAGCGTATCTTCGGGG-3′ for *ZMSPTE*. They were designed with the CRISPR oligo design tool (Feng Zhang). The constructs were made based on the protocol of Ran *et al*.^51^. 48 h after transfection, cells were selected either by culturing cells in the presence of puromycin (1 μg/ml) or by FACS. Control cells underwent the same treatment mentioned above but with a construct containing no gRNA. Individually selected cells were grown to colonies and screened by quantitative immunofluorescence for absence of lamin A/C. Targeting efficiency was validated by high-resolution melt (HRM) analysis around the cut position of the Cas9 protein using the following primers for *LMNA*: forward: 5′-GCATCACCGAGTCTGAAGAG -3′, reverse: 5′-ACTGAGTCAAGGGTCTTGCG -3′ and for *ZMPSTE24*: forward: 5′-CTGGACGCTTTGTGGGAGAT -3′, reverse: 5′-CGCTGTGCTAGGAAGGTCTC -3′. Four of the most abundant potential off-target sites (determined with the CRISPR oligo design tool mentioned above) were amplified and analyzed with Sanger sequencing (Suppl. Table 1). None of these sequences showed a change compared to the control, indicating the specificity of the system.

### Immunofluorescence staining

Cells were fixed with 4% paraformaldehyde for 15 min followed by 3 × 5 min wash steps with PBS (Life technologies, 14190-169). After permeabilisation in 0.5% triton X-100 and blocking for 30 min, primary antibodies were added for 3 h. Cells were either stained with mouse anti-lamin A/C (Santa Cruz, sc-376248, 1/200), rabbit anti-lamin B1 (Abcam, ab16048, 1/1000), goat anti-lamin B (C-20) (Santa cruz, sc6261, 1/250), goat anti-prelamin A (Santa Cruz, sc-6214, 1/150), mouse anti-Nesprin 3 (Mubio, MUB1317S, 1/500)^52^, and mouse anti-Nesprin 2 (K49-260-1, a kind gift from prof. Noegel, University Hospital of Cologne, Germany)^53^. After 3 × 5 min wash steps with PBS, secondary antibodies were added for 1 h. The following secondary antibodies were used: donkey anti-mouse CY3 (Jackson, E00582, 1/1000) and donkey anti-rabbit AF488 (Jackson, E00586, 1/1000). After an additional series of wash steps, DAPI (1 μg/ml) was added for 15 min to the cells and slides were mounted with Vectashield (Vector Labs) or after additional washing the plates were maintained in PBS at 4 °C for microscopy.

### Western blot

Cells were grown in 6-well plates and lysed using the Nucleospin Triprep kit (Macherey-Nagel, 740966). Protein concentration was measured with the Pierce™ BCA Protein Assay Kit (Thermo Scientific, 23227). Cell lysates were subjected to SDS-PAGE (NuPAGE™ Novex™ 4–12% Bis-Tris Protein Gels with MOPS running buffer, Thermo Scientific, J00047) and transferred to BioTrace PVDF membranes (Pall Corporation, 66542). The following primary antibodies were used: mouse anti-lamin A/C (131C3, Santa Cruz Biotechnology Inc., sc-56139, 1/500), mouse anti-lamin A/C (Santa Cruz Biotechnology Inc., sc-376248, 1/200), rabbit anti-CHMP4B (Abcam, ab105767, 1/500) and rabbit anti-nucleolin (Novus Biologicals, NB600-241, 1/4000). HRP conjugated goat anti-mouse (Sigma-Aldrich, A4416) and HRP conjugated goat anti-rabbit (Sigma-Aldrich, A6154) were used as secondary antibodies. Proteins were detected by chemiluminescence with Immobilon Western chemiluminescent HRP substrate (Millipore, WBKLS0100) using a Western Blot Imager (Biorad, ChemiDoc^TM^ XRS+). Bands were automatically detected and quantified with Image Lab^TM^ Software (Biorad).

### qPCR

RNA was extracted using the Nucleospin Triprep kit (Macherey-Nagel). After quality control with the Bioanalyser (Agilent), cDNA synthesis was executed (Tetro cDNA synthesis kit, Bioline) followed by qPCR (Sensimix, Bioline, CFX connect Biorad). Primers were used for *CHMP4B*: forward: 5′-CGAAACCTGTAGGGTTTGGA-3′, reverse: 5′-CTGTTTCGGGTCCACTGATT-3′; *LMNA*: forward: 5′-TGGACGAGTACCAGGAGCTT-3′, reverse: 5′-ACTCCAGTTTGCGCTTTTTG-3′; *ZMPSTE*: forward: 5′-CGAGAAGCGTATCTTCGGGG-3′, reverse: 5′-TGTGCTAGGAAGGTCTCCCA-3′; *ACTB*: forward: 5′-CCTTGCACATGCCGGAG-3′, reverse: 5′-GCACAGAGCCTCGCCTT-3′; *GAPDH*: forward: 5′-TGCACCACCAACTGCTTAGC-3′, reverse: 5′-GGCATGGACTGTGGTCATGAG-3′.

### Microscopy

For live cell imaging, cells were seeded in 4-well glass-bottom dishes (CELLview™, Greiner), and transfected with H2B-GFP and mCherry-NLS 24–48 h prior to imaging. At least three different replicates (i.e. dishes) were used per experiment, and wherever experimental manipulations (compound or siRNA) were used, every dish contained one control and treatment well per cell type (HT-WT and HT-LKO). Time-lapse imaging was performed on a Perkin Elmer Ultraview Vox dual spinning disk confocal microscope, mounted on a Nikon Ti body, equipped with a Perfect Focus System and a microscope incubator equilibrated at 36.5 °C. Recordings were made every 2–3 min, using a 20×/0.75 Plan Achromat dry lens. Image acquisition was done using Volocity software. Per well (condition), 10 regions were monitored, allowing acquisition of 40 different regions in a 2 min time frame. Care was taken to only select cells with moderate expression levels and correct (initial) localization patterns. Cellular condition was also verified by phase contrast microscopy, to assure that cells showed a normal morphology without excessive vacuole formation.

### Image analysis

A dedicated script (trackRuptures.ijm; https://www.uantwerpen.be/en/staff/winnok-devos/cell-systems/scripts/; Suppl. Fig. S1) was written for FIJI image analysis freeware (http://fiji.sc), to automatically track nuclei and measure their signal intensity through time. It runs on images that contain either one single marker (mCherry-NLS) or two markers (mCherry-NLS and H2B-GFP). The analysis workflow is composed of four blocks: image preprocessing, object detection, object tracking and track analysis. Image preprocessing is limited to an intensity normalization step to cover for temporal variations in illumination intensity. Object detection initiates with local contrast enhancement to cover for intercellular variations in fluorescent protein expression level across the field of view. Next, a smoothing step is performed with a 3D (2D + time) kernel, of which the dimensions depend on the construct that is visualized: H2B-GFP images typically only require a small 2D Gaussian filter to reduce noise (lateral smoothing), whereas mCherry-NLS images benefit from a combined lateral and temporal smoothing to buffer the intensity variations due to nuclear ruptures. The temporal smoothing may also be of use for the H2B-GFP signal in case of highly mobile nuclei or focus fluctuations, but can never have too large an extent (max. 10 min); otherwise overt smudging will complicate segmentation. Subsequently, nuclei are detected using a Laplacian of Gaussian blob detector, binarized using an automatic threshold algorithm and touching nuclei are separated using a conditional watershed algorithm which is based on size and intensity criteria. Once nuclei have been detected in all time points, they are connected through time based on a nearest neighbor algorithm, which is confined by a maximum displacement. If for a given nucleus, no corresponding neighbor is found in the next time point, potential candidates are sought in the closest subsequent time point (gap filling). After this automatic procedure, the operator can manually check and correct tracks (manual curation), after which the actual signals are analyzed in one or both channels and listed for further data analysis.

### Statistics

Data analysis and statistics were performed in R Studio, specifically expanded with packages for data structuring (gtools, reshape, plyr), statistics (car, nparcomp), model fitting (diptest, mixtools, fitdistrplus) and visualisation (Hmisc, ggplot2). In brief, raw track analysis output files containing signal intensities per time for every tracked nucleus, were imported in a data frame and cleaned up by removing short tracks (<60 min). The derivative was calculated of the H2B/NLS signal ratio (∆H/N) and rupture events were detected above a threshold value of 1.2 (20% increase). After *in silico* synchronization, parameters such as average rupture frequency and average recovery halftime (T_r_) were statistically compared with parametric (ANOVA) or non-parametric (Dunett or Kruskal Wallis) tests, depending on normality and homoscedasticity checks. Distributions of recovery halftimes were checked for absence of unimodality using Hartigan’s dip test and an optimal fit for the density distributions was obtained by maximum-likelihood and goodness-of-fit estimation via cross-validation or bootstrap sampling.

## Additional Information

**How to cite this article**: Robijns, J. *et al*. *In silico* synchronization reveals regulators of nuclear ruptures in lamin A/C deficient model cells. *Sci. Rep*. **6**, 30325; doi: 10.1038/srep30325 (2016).

## Supplementary Material

Supplementary Information

Supplementary Movie S1

Supplementary Movie S2

Supplementary Movie S3

Supplementary Movie S4
