# Supplementary material for: In silico synchronization reveals regulators of nuclear ruptures in lamin A/C deficient model cells
Source: Sci Rep. 2016 Jul 27;6:30325. doi: 10.1038/srep30325 (PMC4962089; doi:10.1038/srep30325)
Supplement: Supplementary Information [file srep30325-s1.pdf]

SUPPLEMENTARY INFORMATION

***In silico* synchronization reveals regulators of nuclear ruptures in lamin A/C deficient model cells**

J. Robijns[1], F. Molenberghs[1], T. Sieprath[1,2], T. Corne[1,2], M. Verschuuren[1] and W. H. De Vos[1,2*]

[1] Laboratory of Cell Biology and Histology, Department of Veterinary Sciences, University of Antwerp, Antwerp, Belgium

[2] Cell Systems and Imaging Research Group (CSI), Department of Molecular Biotechnology, Ghent University, Ghent, Belgium

Content

- Supplementary Table p. 2
- Supplementary Figures p. 3
- Supplementary Movie Legends p. 11

Supplementary Table 1. Predicted off-target sites for the *LMNA* and *ZMPSTE* knockout gRNA with corresponding primers

		Off Target sequence (5'-3')	Forward primer (5'-3')	Reverse primer (5'-3')
LMNA	1	CTGGAGACTGGGTGATGCGCAGG	TTGTCCAGGTAGGTGGTGGT	GGTGGTGATGTCGCTGTTC
	2	TTGCAGAGTCGGTGATGCTATGG	CGTGCATGGGAGTGTACTTG	ATGTCTGCTGGGGACTGAAT
	3	CCTCAGACAGGATGATGCGATAG	CTCGTCAAGGTAGGGAACTGA	CCCTTCGCTCTTTCTCTCTG
	4	CCACAGACTCACTGATGCGAAGG	ACGGGAAACAACACCATCAG	GCTGAATGCCTAAGCTGGAG
	5	CTTCAGACAGGGTGATGTGAAAG	AGGATGATAGTCCCGAAGCA	GGGAGGAAATCAAATCAGCA
ZMPSTE24	1	GTCTGATAAGCGTATCTTCCCAG	TGCGTCACACTTTAGCATGTC	AACAGAAAGCTGCCGTGACT
	2	GGGTGAGAAGGGTATCTTCCGGG	GTCAGGAAAGCTCAGGCAAG	CACAAAGCGGTCTCTCCACT
	3	GGCAGAGAAACATATCTTCATGG	TTCCAGAGGATGGGTGAGAC	TCTATCGTTGTTGGGCCTTT
	4	GTCCGAGAACAGTATCTTTGTGG	TACCGTGCTTCTGCTGACAT	CAGGAAAGAAACCCCTGACA

Supplementary Figures

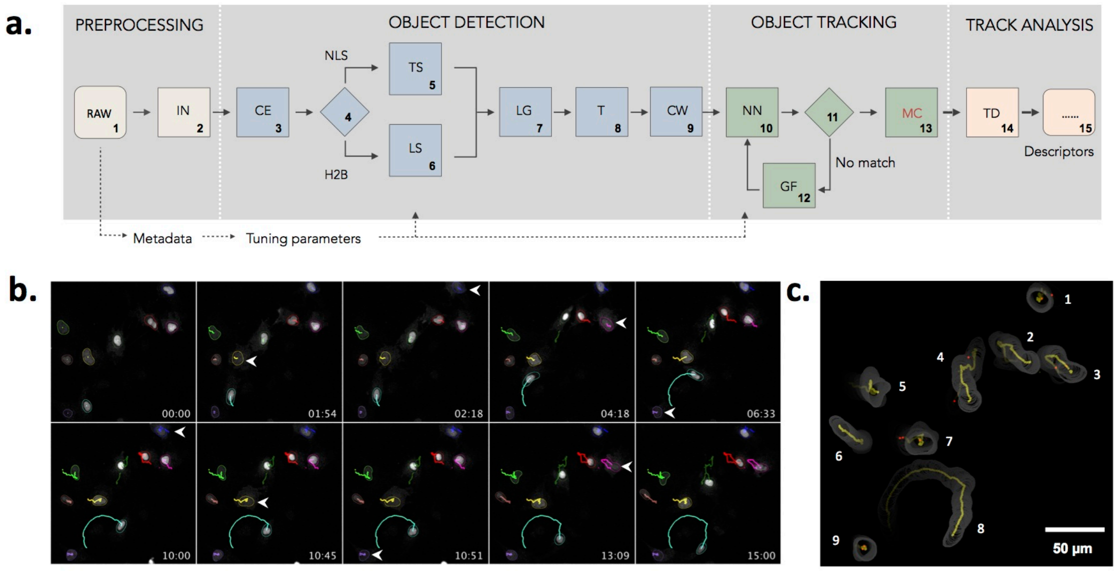

Figure S1. Analysis of spontaneous nuclear ruptures. (a) Image analysis workflow. See Materials and Methods section for more details (IN: intensity normalization, CE: local contrast enhancement, TS: temporal smoothing, LS: lateral smoothing, LG: Laplacian of Gaussian, T: threshold, CW: conditional watershed, NN: nearest neighbor assignment, GF: gap fill, MC: manual curation, TD: track detection); (b) Example result of nuclei tracking in an image data set of MEF-LKO cells, with individually tracked nuclei outlined in distinct colors and superimposed with their resp. tracks. Rupture events are indicated with white arrowheads; (c) Time-projection of the nuclear outlines of the time series in b, superimposed with their respective tracks in yellow. Rupture events are indicated as red dots on the tracks.

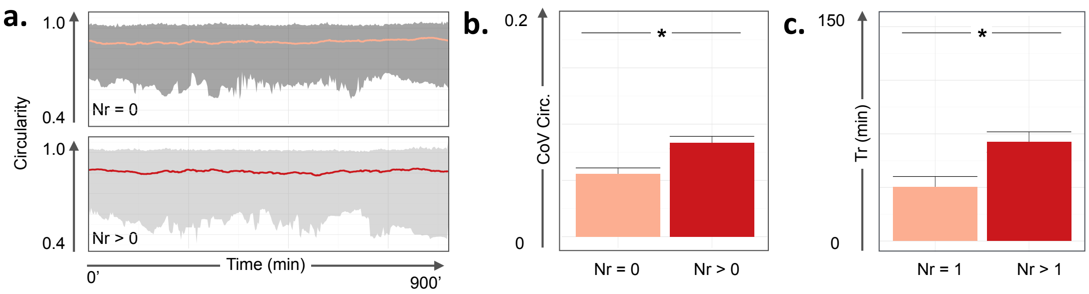

Figure S2. Rupture frequency correlates with nuclear plasticity and repair halftime in MEF-LKO cells (a) MEF-LKO nuclei that experience nuclear ruptures (Nr>0) in a 15 h time span show larger nuclear circularity fluctuations than nuclei that do not (Nr = 0), as shown by the larger range across time (grey); (b) this results in a significantly ($p<0.001$) larger coefficient of variation (CoV) for the circularity across time for rupture-prone cells; (c) The repair halftime is significantly ($p<0.001$) larger for nuclei that experience multiple ruptures (Nr >1), than for nuclei that experience only one rupture event in 15 h. Bar graphs reflect mean ± standard error (n = 218 tracks).

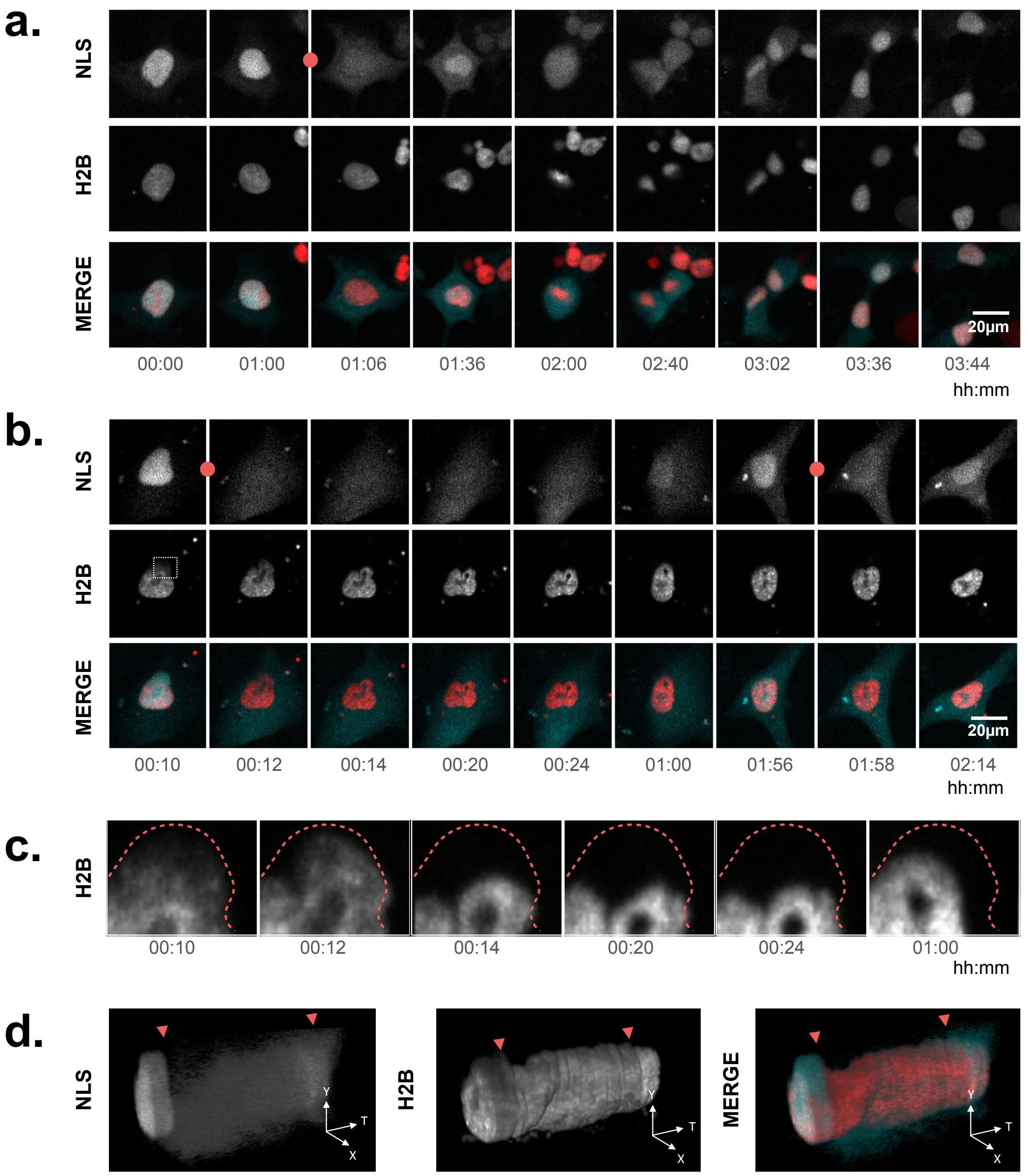

Figure S3. Nuclear ruptures in HT-LKO cells are not lethal and are accompanied by local chromatin condensation (a) Montage of an HT-LKO (L2) cell transfected with H2B-GFP and NLS-mCherry successfully progressing through mitosis after undergoing a rupture event (red dot, 01h:06m). Selected time points of individual channels are shown in grayscale and as merge in red (NLS) and cyan (H2B); (b) Montage of an L2 HT-LKO cell transfected with H2B-GFP (cyan) and NLS-mCherry (red), undergoing repetitive rupture events (red dots) and local chromatin deformation

after the first nuclear rupture; (c) Magnified view of the rectangular region indicated in b superimposed with the outlines of the pre-rupture nuclear shape (00h:10m). Local deformation of the nucleus is accompanied by chromatin condensation, as shown by the concomitant increase in H2B signal intensity; (d) XYT kymographs showing the pronounced deformation after the first nuclear rupture (red arrowhead) and the subsequent local increase in H2B signal intensity reflecting chromatin condensation.

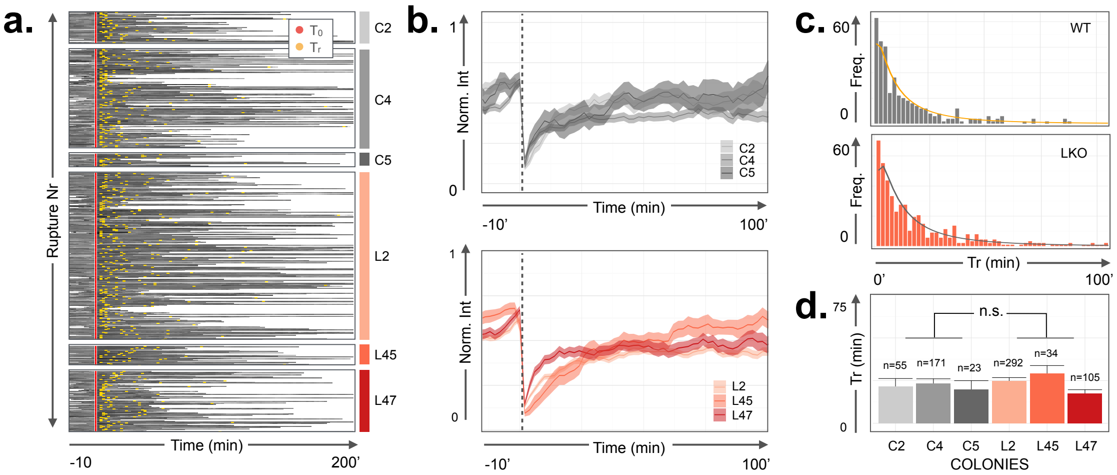

Figure S4. Rupture kinetics of individual HT-WT and HT-LKO clones. (a) Individual rupture events synchronized to the moment of nuclear rupture (red) and marked with recovery halftime (T_r, yellow), grouped per clone. HT-WT clones (C2, C4, C5) are labeled in grey tones and HT-LKO clones (L2, L45, L47) in red tones; (b) Recovery kinetics of nuclear signal after rupture, represented as average signal ± standard error (shaded ribbon). Dotted black line indicates moment of rupture; (c) Frequency histogram of recovery halftimes for HT-WT and HT-LKO cells, superimposed with a lognormal MLE-fitted density distribution; (d) Repair halftimes of individual colonies expressed as mean ± standard error (n = number of tracks).

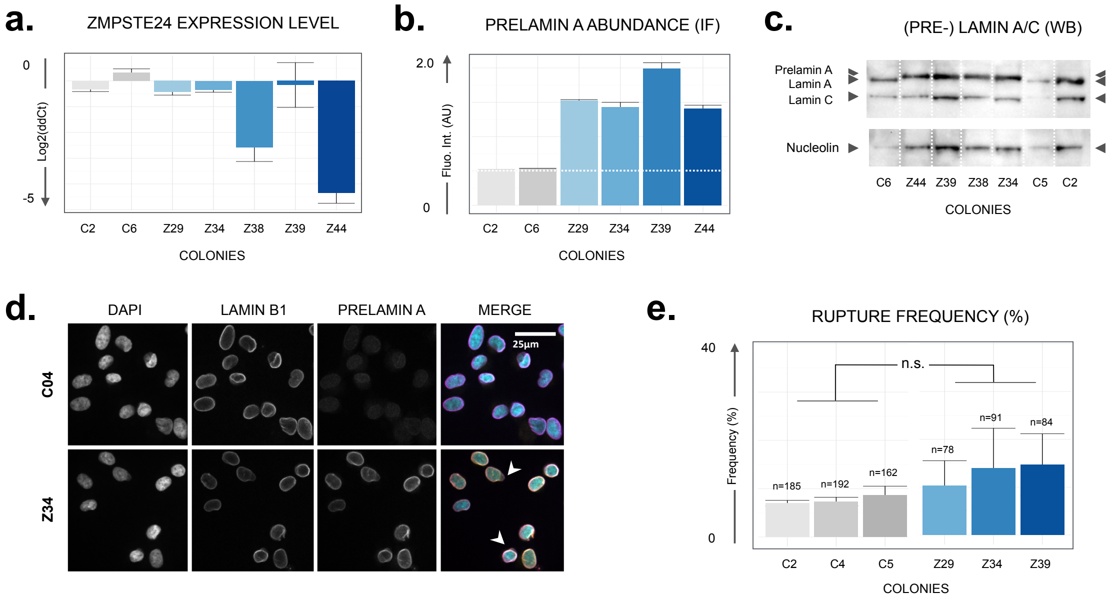

Figure S5. Characterization of HT-ZKO colonies. HT-WT colonies (C2, C4, C5, C6) are labeled in grey tones, and HT-ZKO colonies (Z29, Z34, Z38, Z39, Z44) in blue tones. (a) Quantitative PCR shows variable effects on *ZMPSTE24* expression in individual HT-ZKO colonies; (b) Quantitative immunofluorescence (IF) shows significant accumulation of prelamin A above background levels (dotted white line) in HT-ZKO cells; (c) Western blot (WB) for lamin A/C reveals specific accumulation of prelamin A, and absence of mature lamin A in HT-ZKO cells, as shown by the upward shift of the lamin A band as compared to the HT-WT controls; (d) Immunostaining and nuclear counterstaining of HT-ZKO cells reveals their aberrant nuclear morphology (arrowheads) and accumulation of prelamin A; (e) There is no significant increase in rupture frequency in HT-ZKO cell lines as compared to the HT-WT clones. Bar graphs reflect mean ± standard error (n = number of tracks).

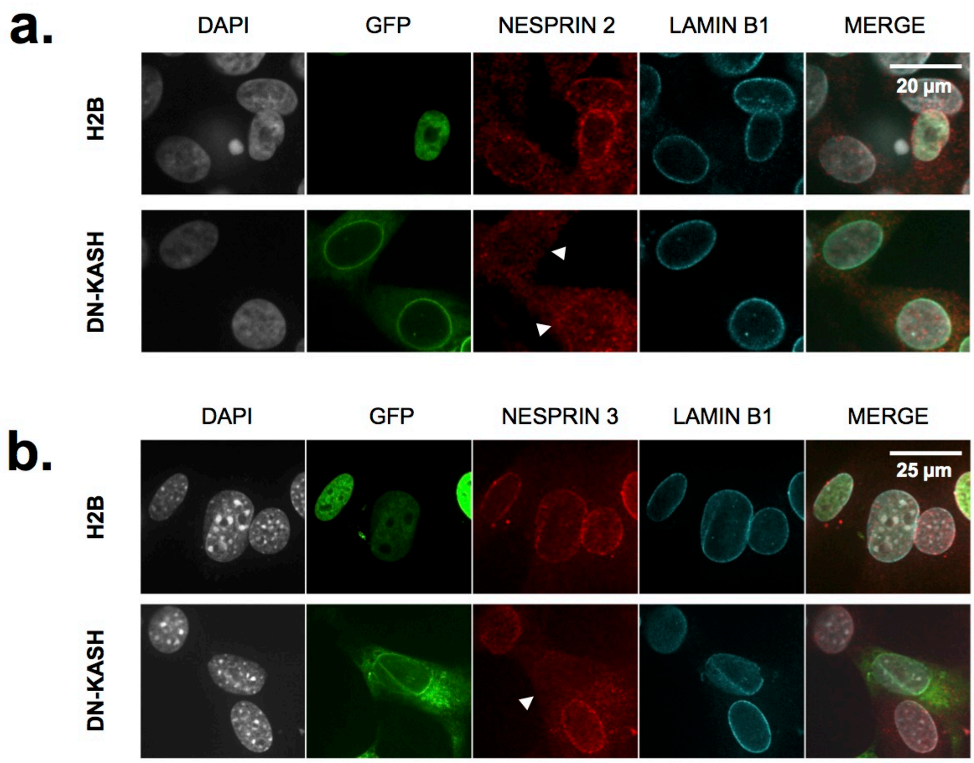

Figure S6. DN-KASH displaces endogenous nesprins from the nuclear envelope. Montages of immunostained HT-WT (a) or MEF-WT (b) cells after transfection with H2B-GFP (top row) or DN-KASH-EGFP (bottom row), respectively. The specific localization of endogenous Nesprin 2 (a) and Nesprin 3 (b) at the nuclear envelope is lost in cells expressing DN-KASH-EGFP (arrowheads), but not in cells expressing H2B-GFP.

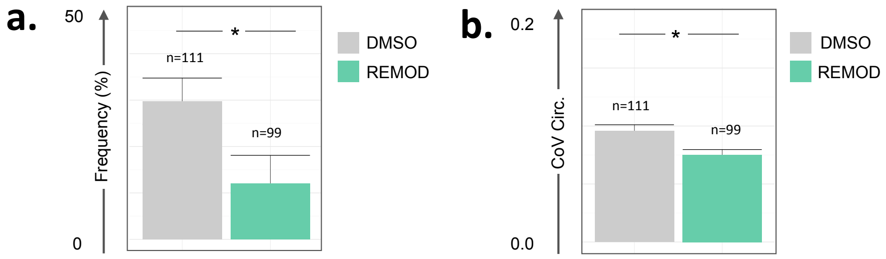

Figure S7. Remodelin treatment reduces rupture frequency in MEF-LKO cells. (a) Remodelin significantly ($p < 0.05$) reduces rupture frequency in MEF-LKO cells; (b) Remodelin treatment significantly ($p < 0.05$) reduces the coefficient of variation of nuclear circularity across time. Bar graphs reflect mean ± standard error (n = number of tracks).

Supplementary Movies

Suppl. Movie 1. Nuclear ruptures in double transfected MEF-LKO cells appear as a strong increase in the derivative signal of the H2B/NLS signal ratio (ΔH/N). Left panel: H2B-GFP; middle panel: mCherry-NLS; right panel: derivative of the H2B/NLS ratio (DEV).

Suppl. Movie 2. Tracking result of MEF-LKO cells undergoing multiple nuclear ruptures. Overlay of the NLS channel and progressive color-coded tracks.

Suppl. Movie 3. Nuclear deformation and local chromatin condensation during rupture in double transfected MEF-LKO cells. Left panel: H2B-GFP; middle panel: mCherry-NLS; right panel: merge of H2B (red) and NLS (cyan).

Suppl. Movie 4. Nuclear rupture in double transfected HT-LKO cells is not lethal. Left panel: mCherry-NLS; middle panel: H2B-GFP; right panel: merge of H2B (red) and NLS (cyan).
